# Recent Advances in Octacalcium Phosphate with Incorporated Carboxylate Ions: A Review

**DOI:** 10.3390/molecules30173508

**Published:** 2025-08-27

**Authors:** Taishi Yokoi, Peng Chen, Masahiro Watanabe, Masakazu Kawashita

**Affiliations:** 1 Laboratory for Biomaterials and Bioengineering, Institute of Integrated Research, Institute of Science Tokyo, 2-3-10 Kanda-Surugadai, Chiyoda-ku, Tokyo 101-0062, Japan; watanabe.masahiro@tmd.ac.jp (M.W.); kawashita.bcr@tmd.ac.jp (M.K.); 2 Division of Interdisciplinary Co-Creation (ICC-Division), Liaison Center for Innovative Dentistry, Graduate School of Dentistry, Tohoku University, 4-1 Seiryo-machi, Aoba-ku, Sendai 980-8575, Japan; peng.chen.b7@tohoku.ac.jp

**Keywords:** octacalcium phosphate, carboxylic acid, octacalcium phosphate carboxylate, functionalisation of octacalcium phosphate, supra-ceramics, artificial bones, bioimaging probes

## Abstract

Octacalcium phosphate (OCP) is a calcium phosphate compound with a layered structure in which apatite layers, which have a structure similar to hydroxyapatite, and hydrated layers are stacked alternately. OCP can incorporate various carboxylate ions into its interlayers. OCPs with incorporated carboxylate ions, also known as OCP carboxylates (OCPCs), are organically modified at the molecular level. OCPCs are an attractive research target in a wide range of fields, from basic inorganic chemistry to applied materials chemistry. Therefore, it is expected that a comprehensive overview of recent research on OCPCs will be useful in progressing this field. This review focuses on recent advances in OCPCs, namely their synthesis, the identification of new types of carboxylate ions that can be incorporated into OCP interlayers, the steric structure estimation of the interlayer carboxylate ions, and applications of OCPCs as functional materials. OCPC-based functional materials include fluorescent materials, artificial bones, and adsorbents. Furthermore, based on existing studies, challenges in OCPC research and future research directions are described.

## 1. Introduction

Phosphoric acid can exist in the form of three dissociated species (H_2_PO_4_^−^, HPO_4_^2−^, and PO_4_^3−^) and therefore reacts with calcium ions in various ratios to form calcium salts. These calcium salts include hydroxyapatite (HAp, Ca_10_(PO_4_)_6_(OH)_2_), tricalcium phosphate (TCP, Ca_3_(PO_4_)_2_), octacalcium phosphate (OCP, Ca_8_(HPO_4_)_2_(PO_4_)_4_·5H_2_O), dicalcium phosphate dihydrate (DCPD, CaHPO_4_·2H_2_O), and dicalcium phosphate anhydrous (CaHPO_4_). In the field of inorganic biomaterials, OCP [1,2,3,4,5,6,7,8,9,10] has long been studied for use in biodegradable artificial bone, along with carbonate apatite [11,12,13,14,15,16,17,18] and calcium carbonate [19,20,21,22,23,24]. The manufacture and sale of artificial bones using a composite material of OCP and collagen were approved in Japan in 2019. OCP is an attractive material not only for medical applications but also from the perspective of inorganic materials chemistry.

OCP has a layered structure in which apatitic layers, which are structurally similar to HAp, and hydrated layers are stacked alternately [25,26,27]. In 1983, Monma and Goto reported that hydrogen phosphate ions (HPO_4_^2−^) in the hydrated layer can be replaced with succinate ions [28]. This property, namely the ability to incorporate carboxylate ions into its crystal, is unique to OCP and not observed in other calcium phosphate compounds. However, not all carboxylic acids can be incorporated into the crystal structure of OCP, and the number of such carboxylic acids is limited [29]. Therefore, the synthesis of OCPs with incorporated carboxylate ions is currently being investigated by several groups. Research on the synthesis of OCPs with incorporated carboxylate ions has been particularly active in Japan, probably because a Japanese researcher, Dr. Monma, discovered the phenomenon of incorporating carboxylate ions into OCP interlayers.

In other countries, for instance, in the United States, Brown et al. synthesised OCPs with incorporated carboxylate ions, structurally estimated the carboxylate ions incorporated between the OCP interlayers [30], and assigned the peaks in their infrared (IR) and Raman spectra [31,32]. In Taiwan, Chan et al. analysed the structure of OCPs with incorporated carboxylate ions using solid-state nuclear magnetic resonance (NMR) spectroscopy [33,34]. While previous solid-state NMR studies have focused on the ^13^C and ^31^P NMR spectra of OCPs with incorporated carboxylate ions, recent research has focused on their ^43^Ca NMR spectra [35].

Forty-two years have passed since the discovery of OCPs with incorporated carboxylate ions, but only around 60 papers in this field have been published so far. This number is extremely small, considering that more than 3000 papers are published each year on HAp, which is a calcium phosphate compound similar to OCP. Therefore, many research areas related to OCPs with incorporated carboxylate ions remain unexplored, compared to HAp. In addition to the development of synthesis methods, the functionalisation of OCPs by incorporating carboxylate ions and their use as precursors for developing novel functional materials has been studied. This review discusses the basic properties and synthesis methods of OCPs with incorporated carboxylate ions, their use in the creation of functional inorganic materials, as well as the challenges and future prospects in this research area, and provides timely and comprehensive information on this material system, in which there has been a resurgence of interest in recent years.

## 2. Incorporation of Carboxylate Ions into OCP Interlayers

Here, we define the OCP-related terms used in this review. Although we prefer to use the expression “OCPs with incorporated carboxylate ions”, the expression “OCP carboxylates” (OCPCs) is also used. For simplicity, the abbreviation “OCPCs” has been used throughout this manuscript. In addition, OCP without carboxylate ions is denoted as “plain OCP”.

### 2.1. Synthesis of OCPCs

#### 2.1.1. Hydrolysis of Calcium Phosphates

The traditional method for synthesising OCPCs involves the transformation of α-TCP into the OCP phase via a dissolution–precipitation reaction in an aqueous solution of the carboxylic acid that is to be incorporated into the OCP interlayers [28,36,37,38,39,40]. The carboxylate ions are incorporated into the OCP interlayers during the formation or crystal growth process, which can be studied using X-ray diffraction (XRD). The replacement of small hydrogen phosphate ions with large carboxylate ions causes an expansion of the crystal plane spacing (specifically, interplanar spacing of the (100) plane, *d*_100_), which can be detected as a peak shift in the XRD pattern.

In addition, a route to synthesise OCPCs using DCPD as a starting material has been proposed [41]. This approach also uses the dissolution–precipitation reaction. However, an advantage of this method is that the calcium ion concentration in the reaction solution is relatively low; therefore, the formation of poorly soluble salts composed of calcium and carboxylate ions can be avoided during their incorporation into the OCP interlayers. Although carboxylate ions can be incorporated into the OCP interlayers by the synthesis method using α-TCP, it is not always possible to incorporate them using DCPD and the reason for this is not yet understood. The fact that the incorporation of a carboxylic acid depends on the synthetic route is interesting in terms of synthetic inorganic chemistry, and it would be meaningful to clarify the underlying reasons. Therefore, this study is significant because it proposes a novel synthetic method and suggests that the incorporation of carboxylate ions into OCP interlayers depends on the synthetic route used.

#### 2.1.2. Synthesis Using Calcium Carbonate and Phosphoric Acid

Kamitakahara et al. proposed a synthetic method for OCPCs in which calcium carbonate and phosphoric acid react in a dicarboxylic acid solution [42]. As separate Ca and P sources are used, the Ca/P molar ratio in this reaction system can be adjusted to that of the target compound, namely the OCPCs (Ca/P = 1.60, stoichiometric composition). This is an important advancement in OCPC synthesis, because the Ca/P molar ratio of the starting materials is fixed when using α-TCP (Ca/P = 1.50) or DCPD (Ca/P = 1.00). Hence, this method improves the compositional flexibility of the reaction system. The authors claimed that the OCPCs synthesised using their method exhibit higher crystallinity than those synthesised using α-TCP. However, the wet method produces plate-shaped OCPC crystals, which are likely to be oriented in samples used for conventional powder XRD analysis. Therefore, the extent to which crystallinity can be evaluated based on XRD results should be considered.

Another advantage of this method is that it produces OCPCs with high carboxylate ion contents. For instance, an OCP with incorporated glutarate ions synthesised using this method showed a high incorporation fraction of 90% [43]. By contrast, the incorporation fraction of an OCPC synthesised using α-TCP was 35% [44]. As the incorporation fraction of OCPCs depends on the synthesis method, exploring different methods for OCPC synthesis is essential.

#### 2.1.3. Homogeneous Precipitation and Co-Precipitation Methods

Homogeneous precipitation and co-precipitation methods are commonly used for synthesising ceramic powders. Although these methods are more limited in number than those discussed so far, OCPC synthesis using these methods has been reported. Notably, OCPs containing amino acids were synthesised by the homogeneous precipitation method using urea hydrolysis [45]. However, the advantages of the homogeneous precipitation method compared to other synthesis methods were unclear in this paper.

**Figure 1:**
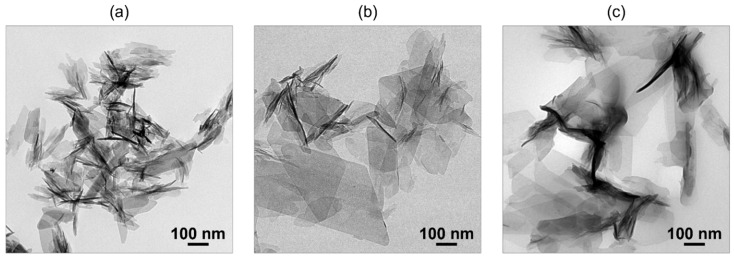
Transmission electron microscopy (TEM) images of (**a**) plain octacalcium phosphate (OCP), (**b**) OCP with incorporated succinate ions, and (**c**) OCP with incorporated isophthalate ions synthesised via co-precipitation. Reprinted from reference [46] with permission from Royal Society of Chemistry, 2024. (Note: The figures have been used after appropriate modification of the original images).

The co-precipitation method is useful for obtaining fine OCPC particles. Based on the LaMer model, a highly supersaturated environment is essential to obtain fine particles. The co-precipitation method is suitable for creating such an environment. In this method, an aqueous calcium acetate solution is mixed with a solution containing ammonium phosphate and dicarboxylic acid to rapidly create a supersaturated environment, and fine OCPC crystals are obtained [46]. Transmission electron microscopy (TEM) images of plain OCP and OCPs with incorporated succinate and isophthalate ions synthesised using this method are shown in Figure 1. Fine particles sized between 100 and 300 nm were obtained using the co-precipitation method.

### 2.2. Carboxylate Ions That Can Be Incorporated into OCP Interlayers

In the 1980s and 1990s, attempts were made to incorporate various carboxylate ions in the OCP interlayers, and the carboxylate ion species that can be incorporated were clarified. A review published in 2022 [29] reports that 25 carboxylic acids can be incorporated into OCP interlayers.

Table 1 lists seven carboxylic acids that have newly been shown to be incorporable into OCPs. Dithiodiglycolic [47] and 3,3′-dithiodipropionic acids [48] contain disulfide bonds in their main chains. OCPs with incorporated dithiodiglycolate ions show a characteristic behaviour in which the dithiodiglycolate ions cause the OCP interlayers to expand due to their decomposition. Of the seven types of dicarboxylic acids in Table 1, four are aromatic dicarboxylic acids [49,50,51]. As mentioned later (Section 3.1), aromatic dicarboxylic acids are now recognised as a major group of carboxylic acids that can be incorporated into OCPs. (Note: Not all aromatic dicarboxylic acids can be incorporated into OCP interlayers.) Interestingly, *meso*-2,3-dimercaptosuccinic acid induces continuous interplanar spacing expansion of the OCPCs [52].

### 2.3. Steric Structure Estimation of Carboxylate Ions in OCP Interlayers

The incorporation of carboxylate ions into OCP interlayers can be determined by XRD, IR spectroscopy, Raman spectroscopy, and solid-state NMR. Among these analytical techniques, XRD is the most powerful tool to study the interplanar spacing expansion that happens when carboxylate ions are incorporated into OCP interlayers.

The carboxylic acids that can be incorporated into the OCP interlayer are mainly dicarboxylic acids. In particular, several straight-chain aliphatic dicarboxylic acids can be incorporated into OCP interlayers. A linear relationship was observed between the interplanar spacing of the (100) planes (*d*_100_) of OCPCs and the size of the incorporated dicarboxylate ions. The incorporation of carboxylate ions causes a clear change in the *d*_100_ of the OCPs, while no systematic change is observed in the *d*_010_ or *d*_002_ [40].

**Figure 2:**
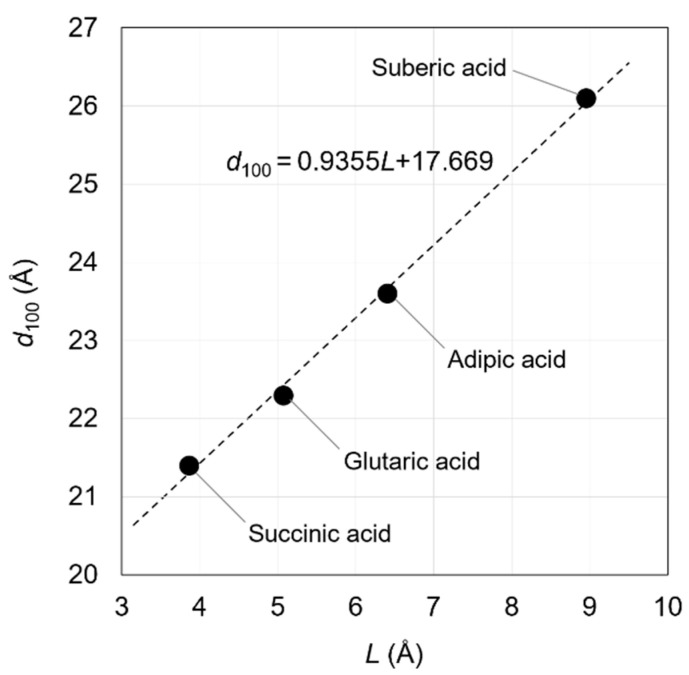
Relationship between *L* (distance between the carbon atoms of the carboxy groups of the carboxylic acids) and the *d*_100_ of OCPs with incorporated succinate, glutarate, adipate, and suberate ions. Reprinted from reference [53] with permission from MDPI, 2021.

This linear relationship holds because the straight-chain aliphatic dicarboxylate ions between OCP interlayers are almost parallel to the *a*-axis of the OCP crystal. This relationship was first reported by Monma in 1984 [40]. In this study, the molecular sizes of dicarboxylic acid molecules were calculated geometrically and with high accuracy, although there were likely some difficulties in determining them. Yokoi et al. calculated molecular sizes of dicarboxylic acids using computational chemistry techniques and established a relationship between the incorporated molecular size and the *d*_100_ value of the OCPCs (Figure 2). This relationship was based on the data of OCPs with incorporated succinate, glutarate, adipate, and suberate ions [53].

*d*_100_ = 0.9355*L* + 17.669 (Å) (1)

In the above equation, the incorporated molecular size (*L*) is defined as the distance between the carbon atoms of the carboxy groups of the carboxylic acids. By experimentally determining *d*_100_, the incorporated molecular size can be estimated using Equation (1). In other words, the steric structures of the molecules between the OCP interlayers can be determined. The relationship between the *d*_100_ of OCPs incorporating straight-chain aliphatic dicarboxylate ions with side chains and their molecular sizes suggests that some types of incorporated carboxylate ions may adopt a bent structure in the OCPCs’ interlayers. Understanding the applicability limits of Yokoi’s equation (Equation (1)) is essential for its appropriate use. This equation hypothesised that the axial angles of the OCP crystal lattice do not change even when carboxylate ions are incorporated into the interlayer. Hence, if the difference between the *d*_100_ predicted from the molecular size and the experimentally obtained *d*_100_ is small, there is likely no need to consider changing the axial angles of the OCP crystal lattice due to incorporation of the carboxylate ions. However, changing the bond angles due to the incorporation of carboxylate ions would be necessary to consider if the difference between the predicted and experimentally obtained *d*_100_ values is large. In this case, it would not be appropriate to predict the conformation of the carboxylate ions in the OCP interlayers based on Yokoi’s equation. In spite of its limitations, Yokoi’s equation is still an effective method for predicting the steric structures of the incorporated carboxylate ions, since no other experimental methods have been reported for the identification of their steric structures.

## 3. Development of Functional Materials Using OCPCs

### 3.1. Fluorescent Materials

In recent years, many fluorescent materials based on OCPCs have been actively investigated. OCP-based fluorescent materials doped with rare earth ions have also been studied, but this review focuses on OCPCs in which fluorescence is caused by the incorporated carboxylate ions. The term “supra-ceramics” has been proposed for materials that exhibit various functions owing to the incorporation of molecular units, including molecular ions [54]. Fluorescent OCPCs are indeed a type of supra-ceramic, a material that follows this cutting-edge research concept.

OCPs containing aromatic dicarboxylic acids, such as phthalic acid derivatives, have been reported previously [44], but at the time, the OCPCs were not considered fluorescent materials. In 2019, the first fluorescent OCPC was reported, in which 2,2′-bipyridine-5,5′-dicarboxylic acid was the source of fluorescence [55]. This carboxylic acid contains two aromatic rings, which are responsible for the fluorescence. This study is significant because it was the first to propose the application of OCPCs as fluorescent materials. Since this discovery, several fluorescent OCPCs have been reported.

**Figure 3:**
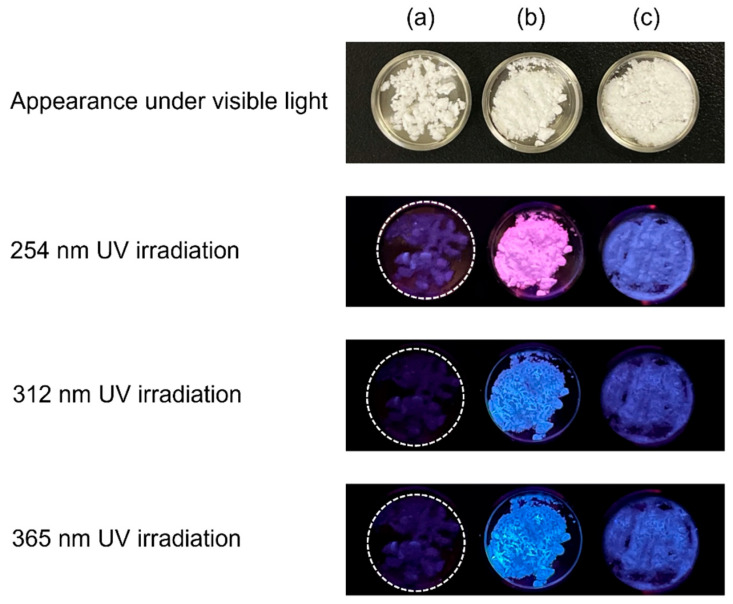
Appearance of (**a**) plain OCP, (**b**) OCP with incorporated 4-(carboxymethyl)benzoate ions, and (**c**) OCP with incorporated *p*-phenylenediacetate ions under visible and ultraviolet (UV) irradiation. Reprinted from reference [49] with permission from the Royal Society of Chemistry, 2024. (Note: The figures have been used after appropriate modification of the original images).

In 2021, the synthesis and fluorescent properties of OCPs with incorporated 1,2,4,5-benzenetetracarboxylate ions were reported [56]. Previously, dicarboxylic acids were the main carboxylic acids that could be incorporated into OCPs, and citric acid was the only reported tricarboxylic acid that could be incorporated into the OCP interlayers. The fact that tetracarboxylic acids can also be incorporated in the OCP interlayer is significant not only for the development of fluorescent materials but also for broadening the range of carboxylic acid species that can be incorporated into OCP interlayers. The three-dimensional fluorescence spectrum of OCP with incorporated 1,2,4,5-benzenetetracarboxylate ions indicated that it was excited by ultraviolet light and emitted blue light at approximately 450 nm. Additionally, fluorescent OCPCs incorporating isophthalic acid [57], 2,5-pyridinedicarboxylic acid [51], 4-(carboxymethyl)benzoic acid, and *p*-phenylenediacetic acid were reported [49]. Of these, the OCP with incorporated 4-(carboxymethyl)benzoate ions is of interest because of its unique fluorescent properties: this OCPC appears pink under 254 nm irradiation and blue under 312 and 365 nm irradiation (Figure 3); at present, this is the only OCPC that exhibits two colours in the visible region. Such fluorescent materials that emit multicoloured light are expected to be useful as bioimaging probes.

OCPC-based materials are expected to have good biocompatibility, and they could potentially be used as bioimaging probes. However, the fluorescent OCPCs obtained by conventional methods are micrometre-sized, plate-shaped particles; therefore, it is difficult to utilize them in fluorescent probes that can be used in cells, such as semiconductor quantum dots [58,59] and rare earth oxide nanoparticles [60,61]. In other words, it is necessary to establish a method for synthesising OCP fine particles. The co-precipitation method described in Section 2.1.3 can be used for synthesising such fluorescent OCPC particles. In this regard, the OCPC particles (OCP with incorporated isophthalate ions, about 300 nm in size) shown in Figure 1c exhibited fluorescent properties [46]. Hence, a promising application of OCPCs is likely next-generation bio-friendly imaging probes.

Here, we briefly mention the future directions for developing bioimaging probes using OCPCs. Currently reported fluorescent OCPCs require ultraviolet light for excitation. Because ultraviolet light can damage living tissues, it is essential to improve OCPC materials so that they can be excited by longer wavelengths. If this limitation is overcome, fluorescent OCPCs could potentially be employed in bioimaging applications.

### 3.2. Artificial Bones

As mentioned in Section 1, plain OCP has long been studied in artificial bone applications. The development of artificial bones using plain OCP has been summarised in the literature [62]. The application of OCPCs in artificial bones was first proposed in 2009 [63]. Because OCPCs cannot be sintered, in this study, OCP with incorporated adipate ions was solidified using hydrothermal-hot pressing, and its apatite-forming ability was investigated using a type of simulated body fluid (SBF). OCPC ceramics prepared by hydrothermal-hot pressing exhibited apatite formation on the material surface. This phenomenon implied that this material is expected to show bone-bonding properties in bone defects. After this study in 2009, investigations into the application of OCP in artificial bones were not carried out for about 10 years, possibly because it was challenging to incorporate effective organic molecules for bone defect healing (such as molecules that promote bone formation) into OCP interlayers.

In recent years, developments in artificial bones using OCPCs, or composite artificial bones prepared using OCPCs as starting materials, have been reported. These studies are described below.

#### 3.2.1. OCP- and OCPC-Based Cement-Type Artificial Bones

In general, plain OCP and OCPCs are obtained in powder form. In practical applications, OCPs are likely to be used as bulk materials. In fact, artificial bones using plain OCP are also bulk composites composed of plain OCP and collagen. Since OCPs contain crystalline water, they dehydrate in high-temperature environments used for ceramic sintering. Furthermore, the hydrogen phosphate ions in OCPs are converted into pyrophosphate ions by a dehydration condensation reaction occurring at high temperatures. Therefore, although OCPs are inorganic materials, they cannot be transformed into bulk materials by sintering.

Recent studies reported that bulk OCP and OCPCs have been prepared using a cement-setting reaction [64,65]. This reaction does not require a heat treatment to obtain bulk materials. In the field of artificial bone research, calcium phosphate cements based on α-TCP and other materials have been studied for many years [66,67] and used clinically [68]. Therefore, it is easy to conceive the idea of producing OCP and OCPCs via a cement-setting reaction for researchers in the ceramic biomaterial field by adjusting the conditions of such reactions.

Bulk OCPs with incorporated succinate and mercaptosuccinate ions were prepared using the cement-setting reaction [64]. The bulk material composed of OCP with incorporated succinate ions, prepared by this method, demonstrated higher diametral tensile strength than that composed of plain OCP. This result is interesting because it highlights an advantage of incorporating carboxylate ions into OCP. However, OCPC-based materials with medicinal effects have not yet been successfully synthesised. In this regard, there is still room for research in the development of artificial bones solely based on OCPCs. Realistically, it may be useful to create superior artificial bones by combining OCPCs with other materials, such as polymers, rather than using them alone.

#### 3.2.2. Composite-Type Artificial Bones

This section describes the development of artificial bones with unique mechanical properties prepared using OCPCs as the starting materials. Ceramic artificial bones are categorised into absorbable and non-absorbable materials. Absorbable materials are replaced by natural bone within several months to a few years after implantation. However, non-absorbable materials remain in the body semi-permanently and hence require mechanical reliability. HAp-sintered bodies are used as non-absorbable artificial bones, but there is a concern that they may break after implantation. To avoid this, non-absorbable artificial bones with excellent resistance to damage are required. However, ceramics are inherently brittle materials, and it is difficult to improve their damage tolerance. In this regard, two studies have reported on the development of highly damage-tolerant artificial bones prepared using OCPCs, and they are discussed below.

**Figure 4:**
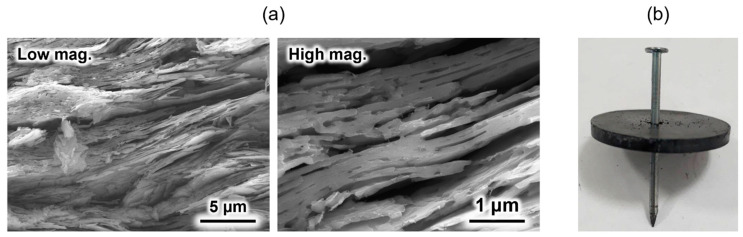
(**a**) Cross-sectional scanning electron microscopy (SEM) images of the HAp/β- tricalcium phosphate (TCP)/pyrolytic carbon composite, and (**b**) post-nailing test image of the composite. Reprinted from reference [69] with permission from Taylor & Francis, 2023. (Note: The figures have been used after appropriate modification of the original images).

An HAp/β-TCP/pyrolytic carbon composite was prepared by sintering a compact composed of OCP with incorporated isophthalate ions in a nitrogen atmosphere [69]. Because the OCP with incorporated isophthalate ions was plate-shaped particles, a compact with a layered structure was spontaneously formed upon pressure moulding. This layered structure remained unchanged even after sintering (Figure 4a). Generally, ceramic-sintered bodies are weak against impact and easily destroyed. However, when a nail was hammered into the sintered body, only the area around the nail was broken, and the entire material did not break (Figure 4b), because the direction of crack propagation when the nail was hammered was deflected by the layered structure. This phenomenon is also observed in natural nacre [70,71] and is often applied in the design of artificial materials [72,73,74] to improve their damage tolerance. In addition, this material was found to be biocompatible in in vitro tests using SBF [69]. Although the crack-propagation control method is conventional, its application to calcium phosphate-based materials for artificial bone applications was reported for the first time. This composite is expected to contribute to the development of novel artificial bones that doctors and patients can use with confidence.

**Figure 5:**
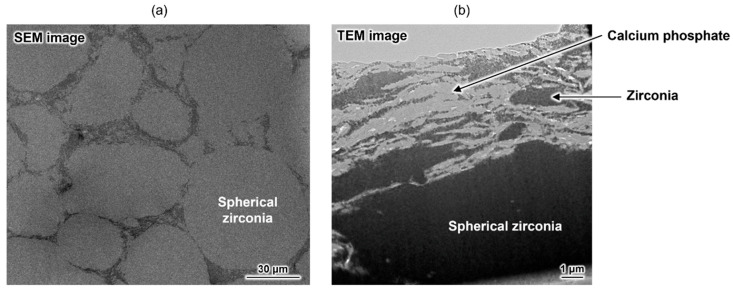
(**a**) Cross-sectional SEM image of the zirconia-based composite and (**b**) TEM image of its grain boundary. Reprinted from reference [75] with permission from American Chemical Society, 2025. (Note: The figures have been used after appropriate modification of the original images).

As mentioned above, a composite with excellent damage tolerance was obtained, but its strength did not reach that of conventional HAp-sintered bodies. To address this issue, an advanced composite was developed by combining zirconia, a known high-strength ceramic material, and the aforementioned calcium phosphate-based material [75]. This material has a unique macroscopic structure, mainly comprising spherical zirconia domains (Figure 5a). Plate-shaped calcium phosphate particles and a small amount of irregularly shaped zirconia particles formed a submicrometre-scaled layered structure at the grain boundary (Figure 5b). The bending strength of the composite was approximately 360 MPa, which is considerably higher than that of conventional HAp-sintered bodies (~100 MPa). This composite also demonstrated unique damage tolerance properties. Therefore, OCPCs can not only be used as functional materials themselves but can also be used for developing new materials with unique functionalities. Thus, this research area is worthy of attention.

### 3.3. Adsorbents

Application of OCPCs in adsorbents was first reported in 2004 [76]. In this study, the adsorption behaviour of aldehydes (gas state) on OCPs with incorporated aspartate ions was investigated. According to IR and solid-state NMR spectra, the amino group of aspartate ions in the OCP interlayers reacted with the aldehydes to form imine compounds, resulting in adsorption. Although the specific surface area of the OCPC was significantly smaller than that of activated charcoal, the adsorption capacity of the OCPC for formaldehyde (3.1 × 10^−3^ mol/g) was considerably higher than that of activated charcoal (1.6 × 10^−3^ mol/g). This study was the first to demonstrate that OCP interlayers can be used as adsorption sites.

In 2025, the adsorption of caffeine into the interlayer space of OCPs with incorporated suberate ions was reported [77]. This study investigated the adsorption of caffeine in the liquid phase and hence differs from the above-mentioned adsorption study on aldehyde gases. In terms of the adsorption mechanism, the adsorption of aldehydes was dominated by the formation of chemical bonds or imine formation. By contrast, the adsorption of caffeine was due to its hydrophobic interactions with the incorporated suberate ions in OCP. These reports suggest that novel adsorbents in gas and liquid phases with well-controlled chemical and physical adsorption properties can be developed using OCPCs.

## 4. Future Challenges in OCPC Research

Two major challenges exist in OCPC research. First, there is considerable room for research in OCPC synthesis. Not all carboxylic acids can be incorporated into OCP interlayers. However, the carboxylate ion selection mechanism of OCPs has not been extensively investigated. It is also unclear why anions other than carboxylate ions cannot be incorporated into OCPs. The incorporation of carboxylate ions in OCP interlayers depends not only on the physicochemical properties of the carboxylate ions but also on the synthesis conditions. Hence, developing novel synthesis processes for OCPCs is essential.

Second, the detailed crystal structure of OCPCs remains unclear. In this review, a method for predicting the steric structure of carboxylate ions in OCP interlayers using Yokoi’s equation was discussed. While this method is significant, clarifying the crystal structure of OCPCs at the atomic coordinate level is essential. No successful experimental crystal structure analyses of OCPCs have yet been reported, and this will be an essential topic for future studies. In addition, quantum computational chemistry techniques can be used to predict the various physicochemical properties of a substance based on its crystal structure. Such techniques may help find novel applications for OCPCs.

## 5. Conclusions

This review not only highlighted the recent achievements in basic OCPC research but also described developments in OCPC-based functional materials. It is expected that novel OCPC-based materials can be developed by incorporating carboxylate ions into OCP interlayers. The search for carboxylic acids that can be incorporated into OCP interlayers and the development of functional materials using OCPCs are two sides of the same coin. The development of fluorescent materials using OCPCs discussed in this review is a good example of this relationship. The development of artificial bones using OCPCs was a major step forward because it revealed that incorporating carboxylate ions into OCP interlayers contributes to improving their mechanical properties. However, OCPs incorporating carboxylic acids that contribute to bone regeneration have not yet been reported, and this challenge must be addressed in future studies. Meanwhile, the approach of developing artificial bones using OCPC as a precursor is novel, and further developments in this area are expected. Interestingly, OCPC can be used as an adsorbent in both the gas and liquid phases. Nevertheless, much about OCPCs remains unknown. For instance, this review also discusses the method for estimating the steric structures of carboxylate ions incorporated into OCP interlayers using Yokoi’s equation. Although this method provides useful information, the experimental steric structure analysis of the incorporated carboxylate ions is essential. Additionally, in the future, it will be necessary to confirm the consistency between the results obtained using Yokoi’s equation and the steric structure of the incorporated carboxylate ions. Therefore, further research is required to clarify the true nature of this fascinating substance. This knowledge will facilitate the development of new revolutionary materials based on OCPCs. We sincerely hope that this review will contribute to the advancement of OCPC research.

## Figures and Tables

**Table 1:** List of carboxylic acids that can be incorporated into OCPs.

Carboxylic Acid (CAS No.)	Formula	Mw *	OCPC Formation	Year
Dithiodiglycolic acid (505-73-7)	HOOCCH_2_SSCH_2_COOH	182.21	Yes	2023
3,3′-Dithiodipropionic acid (1119-62-6)	HOOC(CH_2_)_2_SS(CH_2_)_2_COOH	210.26	Yes	2023
2,5-Pyridinedicarboxylic acid (100-26-5)	HOOC(C_5_H_3_N)COOH	167.12	Yes	2023
2,6-Pyridinedicarboxylic acid (499-83-2)	HOOC(C_5_H_3_N)COOH	167.12	Yes	2024
4-(Carboxymethyl)benzoic acid (501-89-3)	HOOC(C_6_H_4_)CH_2_COOH	180.16	Yes	2024
1,4-Phenylenediacetic acid (7325-46-4)	HOOCCH_2_(C_6_H_4_)CH_2_COOH	194.19	Yes	2024
*meso*-2,3-dimercaptosuccinic acid (304-55-2)	HOOCCH(SH)CH(SH)COOH	182.21	Yes	2024

* Mw: Molecular weight

## Data Availability

No new data were created or analysed in this study.

## Abbreviations

The following abbreviations are used in this manuscript:

HAp	Hydroxyapatite
TCP	Tricalcium phosphate
OCP	Octacalcium phosphate
DCPD	Dicalcium phosphate dihydrate
IR	Infrared
NMR	Nuclear magnetic resonance
OCPC	Octacalcium phosphate carboxylate
XRD	X-ray diffraction
TEM	Transmission electron microscope
UV	Ultraviolet
SBF	Simulated body fluid
SEM	Scanning electron microscope

## References

[1] Sugai Y., Hamai R., Shiwaku Y., Anada T., Tsuchiya K., Kimura T., Tadano M., Yamauchi K., Takahashi T., Egusa H. (2025). Effect of octacalcium phosphate on osteogenic differentiation of induced pluripotent stem cells in a 3D hybrid spheroid culture. Biomimetics.

[2] Hamai R., Tomitsuka K., Okada M., Tsuchiya K., Kimura T., Sakai S., Yamauchi K., Suzuki O. (2025). Osteoclast formation in the co-presence of octacalcium phosphate and inorganic phosphorus-containing gelatin in vitro. Dent. Mater. J..

[3] Xiao L., Shiwaku Y., Hamai R., Baba K., Tsuchiya K., Imazato S., Sasaki K., Suzuki O. (2023). Osteogenic capacity of octacalcium phosphate involving macrophage polarization. J. Biomed. Mater. Res. Part A.

[4] Okuyama K., Shiwaku Y., Hamai R., Mizoguchi T., Tsuchiya K., Takahashi T., Suzuki O. (2022). Differentiation of committed osteoblast progenitors by octacalcium phosphate compared to calcium-deficient hydroxyapatite in Lepr-cre/Tomato mouse tibia. Acta Biomater..

[5] Koyama S., Hamai R., Shiwaku Y., Kurobane T., Tsuchiya K., Takahashi T., Suzuki O. (2022). Angio-osteogenic capacity of octacalcium phosphate co-precipitated with copper gluconate in rat calvaria critical-sized defect. Sci. Technol. Adv. Mater..

[6] Hamai R., Sakai S., Shiwaku Y., Anada T., Tsuchiya K., Ishimoto T., Nakano T., Suzuki O. (2022). Octacalcium phosphate crystals including a higher density dislocation improve its materials osteogenecity. Appl. Mater. Today.

[7] Kibe T., Maeda-Iino A., Takahashi T., Kamakura S., Suzuki O., Nakamura N. (2021). A follow-up study on the clinical outcomes of alveolar reconstruction using octacalcium phosphate granules and atelocollagen complex. J. Oral Maxillofac. Surg..

[8] Kawai T., Kamakura S., Matsui K., Fukuda M., Takano H., Iino M., Ishikawa S., Kawana H., Soma T., Imamura E. (2020). Clinical study of octacalcium phosphate and collagen composite in oral and maxillofacial surgery. J. Tissue Eng..

[9] Kawai T., Suzuki O., Matsui K., Tanuma Y., Takahashi T., Kamakura S. (2017). Octacalcium phosphate collagen composite facilitates bone regeneration of large mandibular bone defect in humans. J. Tissue Eng. Regen. Med..

[10] Kawai T., Echigo S., Matsui K., Tanuma Y., Takahashi T., Suzuki O., Kamakura S. (2014). First clinical application of octacalcium phosphate collagen composite in human bone defect. Tissue Eng. Part A.

[11] Tan J.L.T., Shimabukuro M., Tsuchiya A., Wijekoon W.M.R.L.K., Kishida R., Kawashita M., Ishikawa K. (2025). Influence of porogens on architecture and osteogenesis of porous carbonate apatite artificial bones. Ceram. Int..

[12] Takeda S., Tsuchiya A., Moriyama M., Ishikawa K. (2025). Optimization of the structure of three-dimensional interconnected porous carbonate apatite bone grafts with spherical granules. Dent. Mater. J..

[13] Shibahara K., Hayashi K., Nakashima Y., Ishikawa K. (2025). Controlling the pore size of carbonate apatite honeycomb scaffolds enhances orientation and strength of regenerated bone. Biomater. Adv..

[14] Hayashi K., Teramoto E., Taleb Alashkar A.N., Lou Z., Moriyama M., Ishikawa K. (2025). Antibacterial and angiogenic copper phosphate-modified carbonate apatite honeycomb plugs for enhanced gingival and alveolar bone regeneration. ACS Appl. Mater. Interfaces.

[15] Freitas P., Tan J.L.T., Ishikawa K. (2025). Monolithic copper-doped carbonated apatite synthesized at low-temperature exhibits in vitro antibacterial effect and in vivo bone regenerative properties. Materialia.

[16] Takeda S., Tsuchiya A., Moriyama M., Ishikawa K. (2024). Physical and histological comparison of three-dimensional interconnected porous carbonate apatite bone graft fabricated with cylindrical and spherical granules. Ceram. Int..

[17] Hayashi K., Zhang C., Taleb Alashkar A.N., Ishikawa K. (2024). Carbonate apatite honeycomb scaffold-based drug delivery system for repairing osteoporotic bone defects. ACS Appl. Mater. Interfaces.

[18] Hayashi K., Kishida R., Tsuchiya A., Ishikawa K. (2024). Transformable carbonate apatite chains as a novel type of bone graft. Adv. Healthc. Mater..

[19] Umemoto S., Furusawa T., Unuma H., Goto T., Sekino T. (2025). Evaluation of resorption rate-controlled calcium carbonate ceramics as a substitute bone material. Dent. Mater. J..

[20] Umemoto S., Furusawa T., Unuma H., Tajika M., Sekino T. (2021). In vivo bioresorbability and bone formation ability of sintered highly pure calcium carbonate granules. Dent. Mater. J..

[21] Li X., Yang X., Liu X., He W., Huang Q., Li S., Feng Q. (2018). Calcium carbonate nanoparticles promote osteogenesis compared to adipogenesis in human bone-marrow mesenchymal stem cells. Prog. Nat. Sci. Mater. Int..

[22] Woldetsadik A.D., Sharma S.K., Khapli S., Jagannathan R., Magzoub M. (2017). Hierarchically porous calcium carbonate scaffolds for bone tissue engineering. ACS Biomater. Sci. Eng..

[23] Neumeyer D., Venturini C., Ratel-Ramond N., Verelst M., Gourdon A. (2017). Simple and economic elaboration of high purity CaCO_3_ particles for bone graft applications using a spray pyrolysis technique. J. Mater. Chem. B.

[24] Tolba E., Müller W.E.G., El-Hady B.M.A., Neufurth M., Wurm F., Wang S., Schröder H.C., Wang X. (2016). High biocompatibility and improved osteogenic potential of amorphous calcium carbonate/vaterite. J. Mater. Chem. B.

[25] Mathew M., Brown W.E., Schroeder L.W., Dickens B. (1988). Crystal structure of octacalcium bis(hydrogenphosphate) tetrakis(phosphate) pentahydrate, Ca_8_(HPO_4_)_2_(PO_4_)_4_∙5H_2_O. J. Crystallogr. Spectrosc. Res..

[26] Brown W.E., Smith J.P., Lehr J.R., Frazier A.W. (1962). Octacalcium phosphate and hydroxyapatite: Crystallographic and chemical relations between octacalcium phosphate and hydroxyapatite. Nature.

[27] Brown W.E., Lehr J.R., Smith J.P., Frazier A.W. (1957). Crystallography of octacalcium phosphate. J. Am. Chem. Soc..

[28] Monma H., Goto M. (1983). Succinate-complexed octacalcium phosphate. Bull. Chem. Soc. Jpn..

[29] Yokoi T., Shimabukuro M., Kawashita M. (2022). Octacalcium phosphate with incorporated carboxylate ions: A review. Sci. Technol. Adv. Mater..

[30] Mathew M., Brown W.E. (1987). A structural model for octacalcium phosphate-succinate double salt. Bull. Chem. Soc. Jpn..

[31] Fowler B.O., Marković M., Brown W.E. (1993). Octacalcium phosphate. 3. Infrared and Raman vibrational spectra. Chem. Mater..

[32] Marković M., Fowler B.O., Brown W.E. (1993). Octacalcium phosphate carboxylates. 2. Characterization and structural considerations. Chem. Mater..

[33] Li Y., Reid D.G., Duer M.J., Chan J.C.C. (2018). Solid state NMR–An indispensable tool in organic-inorganic biocomposite characterization; refining the structure of octacalcium phosphate composites with the linear metabolic di-acids succinate and adipate. Solid State Nucl. Magn. Reson..

[34] Tsai T.W.T., Chou F.-C., Tseng Y.-H., Chan J.C.C. (2010). Solid-state P-31 NMR study of octacalcium phosphate incorporated with succinate. Phys. Chem. Chem. Phys..

[35] Laurencin D., Li Y., Duer M.J., Iuga D., Gervais C., Bonhomme C. (2021). A ^43^Ca nuclear magnetic resonance perspective on octacalcium phosphate and its hybrid derivatives. Magn. Reson. Chem..

[36] Nakahira A., Aoki S., Sakamoto K., Yamaguchi S. (2001). Synthesis and evaluation of various layered octacalcium phosphates by wet-chemical processing. J. Mater. Sci. Mater. Med..

[37] Aoki S., Sakamoto K., Yamaguchi S., Nakahira A. (2000). Syntheses of octacalcium phosphate containing dicarboxylic acids and effects of the side groups on the crystal growth of octacalcium phosphate. J. Ceram. Soc. Jpn..

[38] Marković M., Fowler B.O., Brown W.E. (1993). Octacalcium phosphate carboxylates. 1. Preparation and identification. Chem. Mater..

[39] Monma H., Nishikawa H. (1992). Thermal behavior of octacalcium phosphate intercalated with *β*-dihydromuconate. J. Ceram. Soc. Jpn..

[40] Monma H. (1984). The incorporation of dicarboxylates into octacalcium bis(hydrogenphosphate) tetrakis(phosphate) pentahydrate. Bull. Chem. Soc. Jpn..

[41] Yokoi T., Goto T., Kitaoka S. (2018). Transformation of dicalcium phosphate dihydrate into octacalcium phosphate with incorporated dicarboxylate ions. J. Ceram. Soc. Jpn..

[42] Kamitakahara M., Okano H., Tanihara M., Ohtsuki C. (2008). Synthesis of octacalcium phosphate intercalated with dicarboxylate ions from calcium carbonate and phosphoric acid. J. Ceram. Soc. Jpn..

[43] Yokoi T., Watanabe M., Goto T., Meng S., Sekino T., Shimabukuro M., Kawashita M. (2022). Synthesis of octacalcium phosphate containing glutarate ions with a high incorporation fraction. Materials.

[44] Monma H. (1992). Apatitic intercalation compounds containing dicarboxylates. Gypsum Lime.

[45] Kijima T., Yamaguchi K., Miyata A., Yada M., Machida M., Tanaka J. (2000). Crystallization of calcium phosphate templated by α-amino acids depending on their composition, chain length, and enantiomerism. Chem. Lett..

[46] Yokoi T., Goto T., Sekino T., Hasegawa T., Chen P., Kanetaka H., Yoshida K., Shimabukuro M., Kawashita M. (2024). LaMer-model-based synthesis method for fine particles of octacalcium phosphate and related functional compounds. CrystEngComm.

[47] Sugiura Y., Yamada E., Horie M. (2023). Interlayer expansion of octacalcium phosphate via forced oxidation of the intercalated molecules within its interlayers. Phys. Chem. Chem. Phys..

[48] Yokoi T., Watanabe M., Nakamura F., Kimura-Suda H., Shimabukuro M., Kawashita M. (2023). Formation of octacalcium phosphate with incorporated dicarboxylate ions containing disulfide bonds. Dalton Trans..

[49] Yokoi T., Watanabe M., Kawashita M. (2024). Octacalcium phosphate with incorporated terephthalate ion derivatives: Novel guest molecules and unique fluorescence properties. Dalton Trans..

[50] Kataoka T., Nishiyama S., Fujii E., Yoshioka T., Hayakawa S. (2024). Synthesis and structural characterization of Sr(II) ion-containing octacalcium phosphate incorporated with both 2,6-pyridinedicarboxylate and succinate ions as a bone defect filler with multiple drug loading capability. Colloids Surf. A Physicochem. Eng. Aspects.

[51] Yokoi T., Watanabe M., Wang Y., Goto T., Sekino T., Shimabukuro M., Kawashita M. (2023). Synthesis and fluorescence properties of octacalcium phosphate with incorporated pyridinedicarboxylate ions. J. Ceram. Soc. Jpn..

[52] Yokoi T., Watanabe M., Kawashita M. (2024). Octacalcium phosphate with incorporated *meso*-2,3-dimercaptosuccinate ions exhibiting continuous interplanar spacing expansion. J. Ceram. Soc. Jpn..

[53] Yokoi T., Kawashita M. (2021). Understanding the steric structures of dicarboxylate ions incorporated in octacalcium phosphate crystals. Materials.

[54] Maeda K., Motohashi T., Ohtani R., Sugimoto K., Tsuji Y., Kuwabara A., Horike S. (2024). Supra-ceramics: A molecule-driven frontier of inorganic materials. Sci. Technol. Adv. Mater..

[55] Yamada I., Tagaya M. (2019). Immobilization of 2,2′-bipyridine-5,5′-dicarboxylic acid in layered octacalcium phosphate. Colloids Interface Sci. Commun..

[56] Yokoi T., Goto T., Hara M., Sekino T., Seki T., Kamitakahara M., Ohtsuki C., Kitaoka S., Takahashi S., Kawashita M. (2021). Incorporation of tetracarboxylate ions into octacalcium phosphate for the development of next-generation biofriendly materials. Commun. Chem..

[57] Yokoi T., Goto T., Sekino T., Kawashita M. (2022). Fluorescent properties of octacalcium phosphate with incorporated isophthalate ions. J. Ceram. Soc. Jpn..

[58] Bandaru S., Palanivel M., Ravipati M., Wu W.-Y., Zahid S., Halkarni S.S., Dalapati G.K., Ghosh K.K., Gulyás B., Padmanabhan P. (2024). Highly monodisperse, size tunable glucosamine conjugated CdSe quantum dots for enhanced cellular uptake and bioimaging. ACS Omega.

[59] Zhang H., Sun C., Sun L., Xu W., Wu W., Chen J., Wang B., Yu J., Cui P., Zhang F. (2022). Stable monodisperse Pb_1−*x*_Cd*_x_*S quantum dots for NIR-II bioimaging by aqueous coprecipitation of bimetallic clusters. Angew. Chem. Int. Ed..

[60] Venkatachalam N., Yamano T., Hemmer E., Hyodo H., Kishimoto H., Soga K. (2013). Er^3+^-doped Y_2_O_3_ nanophosphors for near-infrared fluorescence bioimaging applications. J. Am. Ceram. Soc..

[61] Hemmer E., Yamano T., Kishimoto H., Venkatachalam N., Hyodo H., Soga K. (2013). Cytotoxic aspects of gadolinium oxide nanostructures for up-conversion and NIR bioimaging. Acta Biomater..

[62] Insley G., Suzuki O. (2019). Octacalcium Phosphate Biomaterials: Understanding of Bioactive Properties and Application.

[63] Ishihara S., Matsumoto T., Onoki T., Sohmura T., Nakahira A. (2009). New concept bioceramics composed of octacalcium phosphate (OCP) and dicarboxylic acid-intercalated OCP via hydrothermal hot-pressing. Mater. Sci. Eng. C.

[64] Sugiura Y., Saito Y., Yamada E., Horie M. (2024). Fabrication of dicarboxylic-acid- and silica-substituted octacalcium phosphate blocks with stronger mechanical strength. Ceramics.

[65] Sugiura Y., Yamada E., Horie M. (2023). Fabrication of dicarboxylic-acid-substituted octacalcium phosphate blocks via cementing. Ceram. Int..

[66] Bermúdez O., Boltong M.G., Driessens F.C.M., Planell J.A. (1994). Development of some calcium phosphate cements from combinations of α-TCP, MCPM and CaO. J. Mater. Sci. Mater. Med..

[67] Mirtchi A.A., Lemaitre J., Terao N. (1989). Calcium-phosphate cements: Study of the beta-tricalcium phosphate–monocalcium phosphate system. Biomaterials.

[68] Matsumine A., Kusuzaki K., Matsubara T., Okamura A., Okuyama N., Miyazaki S., Shintani K., Uchida A. (2006). Calcium phosphate cement in musculoskeletal tumor surgery. J. Surg. Oncol..

[69] Kuroyama K., Fujikawa R., Goto T., Sekino T., Nakamura F., Kimura-Suda H., Chen P., Kanetaka H., Hasegawa T., Yoshida K. (2023). Development of bioinspired damage-tolerant calcium phosphate bulk materials. Sci. Technol. Adv. Mater..

[70] Kakisawa H., Sumitomo T. (2011). The toughening mechanism of nacre and structural materials inspired by nacre. Sci. Technol. Adv. Mater..

[71] Barthelat F., Espinosa H.D. (2007). An experimental investigation of deformation and fracture of nacre–mother of pearl. Exp. Mech..

[72] Wang Y., Liu Q., Zhang B., Zhang H., Zhong Z., Ye J., Ren Y., Shen L., Ye F., Wang W. (2022). Bioinspired nacre-like 2024Al/B4C composites with high damage tolerance. Ceram. Int..

[73] Wan H., Leung N., Jargalsaikhan U., Ho E., Wang C., Liu Q., Peng H.-X., Su B., Sui T. (2022). Fabrication and characterisation of alumina/aluminium composite materials with a nacre-like micro-layered architecture. Mater. Des..

[74] Li Y.-L., Guo R.-F., Hu Z.-J., Shen P. (2021). Construction of nacre-mimetic composites with a “brick-and-mortar” architecture based on structural defects in ice-templating. Mater. Des..

[75] Yokoi T., Chen P., Yoshida K., Seo Y., Goto T., Kuroyama K., Sekino T., Hasegawa T., Yoda T., Kanetaka H. (2025). Development of zirconia/calcium phosphate/pyrolytic carbon composites with nanoscale lamellar-structured grain boundary phases to control crack propagation for biomedical applications. ACS Appl. Nano Mater..

[76] Aoki S., Nakahira A., Nakayama H., Sakamoto K., Yamaguchi S., Suganuma K. (2004). Synthesis and aldehyde absorption properties of aspartate-octacalcium phosphate inclusion compound. J. Phys. Chem. Solids.

[77] Kataoka T., Koga A., Nishiyama S., Yoshioka T., Hayakawa S. (2025). Improvement of caffeine adsorption on octacalcium phosphate through suberate ion incorporation. Mater. Lett..

